# Tourette Syndrome research highlights 2014

**DOI:** 10.12688/f1000research.6209.2

**Published:** 2015-07-14

**Authors:** Cheryl A Richards, Kevin J Black

**Affiliations:** 1 Department of Psychiatry, Washington University School of Medicine, St. Louis, MO, USA; 2 Departments of Psychiatry, Neurology, Radiology, and Anatomy & Neurobiology, Washington University School of Medicine, St. Louis, MO, USA

**Keywords:** review, histamine, animal models, premonitory urge, MRI, treatment, remission, inheritance

## Abstract

About 200 journal articles reported research on Tourette syndrome and other tic disorders in 2014. Here we briefly summarize a few of the reports that seemed most important or interesting, ranging from animal models to human studies. Readers can comment on our choices or provide their own favorites using the tools on the online article.

## Introduction

**Figure 1.**
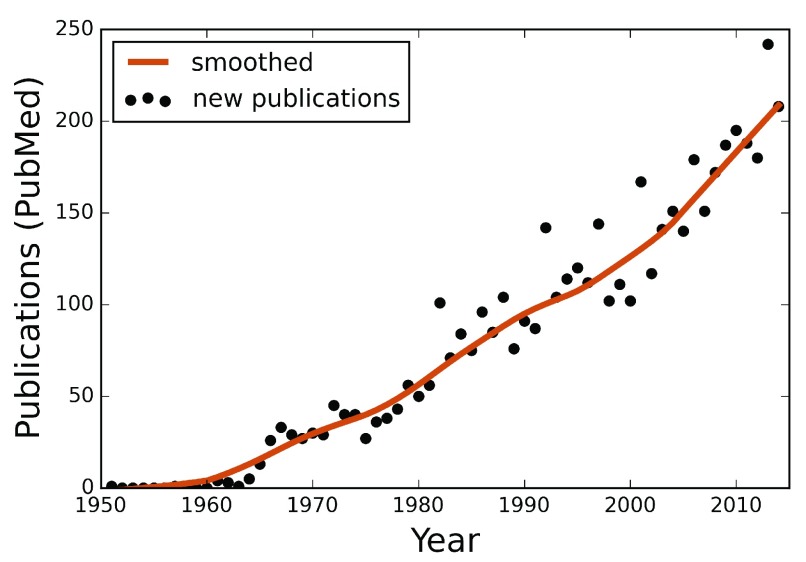
Publications on Tourette syndrome. The number of new publications on Tourette syndrome or other tic disorders each year was estimated from PubMed. The colored line represents locally weighted scatterplot smoothing (LOWESS) of the primary data. PubMed was searched using the search string “(“Tic Disorders”[MeSH] OR Tourette NOT Tourette[AU]) AND *year*[PDAT] NOT *year+1*[PDAT]” for each year from 1950 through 2014. (This strategy assigns articles to the year in which the final publication appeared, to prevent double-counting “early online” and final publication dates for about 250 publications since 2005). The graph was generated by matplotlib in python (see Supplementary material).

The available information on Tourette syndrome (TS) is steadily increasing ( Figure 1), and keeping up with the published literature is therefore an increasing challenge. This article introduces a Highlights article to the *F1000Research: Tics* channel, to showcase some of the most noteworthy publications from the previous calendar year.

## Methods

This article is not a systematic review but summarizes the authors’ personal views. We used the following approach to identify pertinent publications: personal reading, asking colleagues for suggestions, F1000Prime, and a PubMed search ( Figure 1, legend). The search string “("Tic Disorders"[MeSH] OR Tourette NOT Tourette[AU]) AND 2014[PDAT] NOT 2015[PDAT]” produced 201 references (one of which was actually published in 2013). Of course this approach will miss some TS-related publications that do not appear in PubMed, or that will be indexed in PubMed in coming months. We further limited the scope to articles written in English that had final publication dates in 2014.

Publications on Tourette syndrome: 1950–2014 This file contains the PubMed data used to generate Figure 1 in the associated article. Click here for additional data file. Copyright: © 2015 Richards CA and Black KJ 2015 Data associated with the article are available under the terms of the Creative Commons Zero "No rights reserved" data waiver (CC0 1.0 Public domain dedication).

## Results

Here we present examples of TS research published in 2014 that we thought had notable findings or the potential to stimulate additional research in TS.

### Etiology

***A genetic clue.*** To this point, the highly heritable nature of TS has remained a tantalizing clue rather than the key to understanding pathophysiology. However, recently an international collaboration reported an intriguing result. A recent genome-wide association study had identified a number of single nucleotide polymorphisms (SNPs) as possibly associated with TS. The group genotyped 42 of these SNPs in over 1200 individuals, half from unrelated TS cases and half from controls matched for ancestry ^69^. A risk score based on each individual’s alleles at the 42 SNPs was able to predict diagnosis significantly better than chance; this result supports the conclusion that at least some of these SNPs are true risk alleles for TS. One of the SNPs remained significant after correction for multiple comparisons, and the authors discuss nearby genes that could produce relevant changes in brain structure or function.

### Pathophysiology

***Mice without histamine.*** Castellan Baldan *et al.* ^1, 2^ report on characterization of a possible animal model for Tourette syndrome, chosen because of a family with Tourette syndrome linked to a loss-of-function mutation in the histidine decarboxylase gene ^3, 4^. Histidine decarboxylase knock-out mice ^5^ exhibited tic-like stereotypies after a d-amphetamine challenge (see Figure 1, panel E in ref. 2) and increased striatal dopamine during the nocturnal (awake) period. The amphetamine-induced stereotypies were decreased by administration of the dopamine D2 receptor antagonist haloperidol, and histamine infusion reduced striatal dopamine levels and amphetamine-induced stereotypies. Prepulse inhibition and dopamine D2/D3 receptor binding were altered in both the knock-out mice and the small sample of known human carriers of the histidine decarboxylase mutation.

***Other animal models.*** Appropriate animal models of TS could provide pathophysiological insights or speed identification and development of novel treatments. Two informative reviews of mouse models for tic disorders were published in 2014 ^6, 7^. Both reviews agreed that animal models need appropriate face, constructive, and predictive validity to be useful, but Godar *et al.* argue that a focus on intermediate phenotypes, which involve more elemental neuroanatomic and functional deficits, results in animal models with specific measurable parameters ^6^. They then describe several lines of knockout mutant mice using candidate genes for TS, animal models examining the link between early neuroinflammation and TS pathogenesis, and pharmacological models. Pappas *et al.* include mouse models for Rett’s syndrome and primary dystonia in addition to TS/OCD ^7^. They developed a test battery to characterize variations across mouse strains in terms of putative tic-like symptoms (i.e., head twitches and body jerks induced by administration of a selective 5-HT2 receptor agonist), amphetamine-induced stereotypies (i.e., wall-rearing and head-down sniffing), perseverative responding on an attentional set-shifting task involving binary choice, and spontaneous locomotion ^8^. Although DOI administration produced head twitches and body jerks in all subjects, the SJL and C57 mice spent a longer time performing body jerks than the ABH and CD1 mouse strains, with the C57 mice also showing increased elevated levels of spontaneous locomotion and increased perseveration on the set-shifting task. The authors argue that using specific behavioral parameters to investigate differences among mouse strains will allow identification of strains that may represent “pure” TS while other strains may represent TS along with behaviors that reflect common co-morbidities (e.g., hyperactivity, compulsions).

At the November, 2014, annual meeting of the Society for Neuroscience, Xu *et al.* presented evidence that removing about half of the cholinergic interneurons in the dorsolateral striatum reproduced some features of TS in a rodent model ^9^; see also ^10^. This model was inspired by the fascinating autopsy studies that found decreased numbers of striatal interneurons in Tourette syndrome ^11^. At the same meeting, McCairn and colleagues presented a non-human primate model with the GABA antagonist bicuculline injected into the putamen or nucleus accumbens; this produced a variety of movements and vocalizations, including some tics with reasonable face validity ^12^. As the validity of animal models increases, so does the expectation that they may provide additional insights concerning TS and its associated comorbidities.

***A look inside: neuroimaging studies.*** Neuner *et al.* ^13^ examined tic-related neural activity in ten adults with TS using fMRI, estimating the timing of brain activity (as reflected by BOLD signal) to a resolution shorter than that of the individual image acquisitions by taking advantage of the essentially random temporal distribution of tics with respect to the timing of image acquisition. This strategy is reminiscent of the event-related analysis of positron emission tomography regional brain blood flow images developed by Silbersweig and colleagues ^14^. Two seconds before tic occurrence, BOLD activity increased in the supplementary motor area (SMA), ventral primary motor cortex, primary sensorimotor cortex and parietal operculum. One second before tics, activation was seen in the anterior cingulate, putamen, insula, amygdala, cerebellum and extrastriatal visual cortex, and at tic onset activation was seen in the primary motor and somatosensory cortices, the thalamus, and the central operculum. Cortical structure BOLD signal clearly preceded signal in subcortical structures. In addition, resting state data demonstrated that network strength in the same SMA regions correlated with ratings of recent tic severity; the authors suggest that abnormal baseline activity in the SMA may contribute to tic generation. Diffusion tensor imaging identified lower connectivity values (CI), consistent with altered white matter structure, in almost two-thirds of the tracts examined in 15 adults with “pure” TS compared to healthy controls ^15^. After correction for multiple comparisons, 10 tracts were identified as having significantly lower CI in the patient group. Two of these involved connections between the SMA or preSMA and pallidum, and were excluded from further analysis. Correlations between YGTSS scores and individual tract CI values were found for the M1–OFC and preSMA–putamen, although none of these remained significant after correcting for multiple comparisons.

In another fMRI study, adults with “pure” TS performed similarly to control subjects on a stop signal reaction time task, consistent with the conclusion that tics occur without broadly insufficient action inhibition ^16^. However, TS subjects exhibited greater dorsal premotor activation during the Go condition compared to the StopSuccess condition. Increased right pre-SMA activation was associated with successful stop trials in healthy controls. For TS subjects, activation in the SMA proper during StopSuccess compared to Go trials was positively correlated with motor tic frequency. The involvement of SMA in both proactive and reactive control was discussed and the authors suggest that greater SMA activation in patients with higher tic frequency may reflect a stronger need for tic inhibition.

An overview of SMA syndromes and related research explores how the SMA may be involved in both initiation and inhibition of movements along with providing a tonic interhemispheric balance ^17^. This concept of interhemispheric balance may explain why both SMA activation and SMA inhibition can reduce tics, and may also explain why SMA activation can produce echophenomena in healthy controls.

A derived measure called regional homogeneity (“ReHo”) in the left inferior frontal gyrus increased in 14 subjects with pure TS during tic suppression compared to free ticcing ^18^. ReHo increases were positively correlated with participants’ ability to inhibit their tics both inside and outside the scanner. In another report from the same group, grey matter volumes in the right inferior frontal gyrus and left frontal pole were reduced in adults with “pure” TS compared to healthy controls but these reductions were not correlated with Yale Global Tic Severity Scale (YGTSS) scores or the ability to inhibit tics ^19^.

GABA concentrations in the SMA, measured using magnetic resonance spectroscopy, were significantly higher in 15 adolescents with TS compared to 14 age- and gender-matched controls; there were no group differences for GABA concentrations within M1 or primary visual cortex ^20^. The fMRI BOLD signal change within SMA was negatively correlated with SMA GABA levels supporting the idea that Magnetic Resonance Spectroscopy (MRS)-GABA concentrations are associated with localized increases in tonic inhibition. In a small subset of TS subjects, single-pulse transcranial magnetic stimulation (TMS) delivered to the hand area of the left M1 region preceding movement of the right hand revealed a significant negative correlation between MRS-GABA in the SMA and cortical-spinal excitability within the left M1. Fractional anisotropy values within the corpus callosum for TS subjects were positively correlated with the SMA GABA values and with motor tic severity. These authors suggest that enhanced control over volitional movements and tic suppression may be the result of increased tonic inhibition due to the localized increases in extracellular GABA within SMA.

Eight adult subjects who completed Comprehensive Behavioral Intervention for Tics (CBIT) treatment were compared with matched controls on a visual priming task that was used to measure response inhibition ^21^. No significant between-group differences were found in task-related BOLD signal in regions of interest (putamen, caudate, and prefrontal cortex regions BA 11, 44 and 47) before or after CBIT training (with retesting over a comparable amount of time for controls). However, there was a significant group by time interaction because putamen activation decreased in the TS subjects from time 1 to time 2 while it increased in the control subjects. A significant negative correlation between change in inferior frontal gyrus activation and change in YGTSS Total Tic Scores was also found. The authors point out that this result is somewhat difficult to interpret given that prior research has indicated that frontal regions are involved in tic suppression.

***Caveats...*** Several groups have recently studied the substantial effects small head motions can have on BOLD fMRI. It is becoming apparent that many of the established techniques to control for movement effects are frequently not sufficient. Functional connectivity analyses have been especially affected, since small head movements during scanning can produce artifactual connectivity signal (i.e., bias not just noise). Fortunately, robust methods exist for preventing such artifact, at the cost of potentially longer acquisition times ^22^. However, movement also interferes with task fMRI analysis. In one task fMRI study of 73 TS subjects ranging in age from 9–15 years of age, only 38 subjects remained after excluding subjects with less than 70% accuracy on a rule-switching task and with at least 3 runs out of 6 with root-mean-square head movement estimates below 1.5 mm ^23^. This is not all explained by tics, since 33 of 53 healthy, tic-free children aged 7–9 were excluded for task accuracy <60% or head motion > 1.5 mm (rms). Even with these relatively stringent requirements, frame-by-frame motion censoring excluded an additional 15–20% of the data. However, this approach bought cleaner signal; motion censoring performed better than all forms of motion regression ^23^.

Minor head motion has been shown to affect structural brain imaging as well. Diffusion MRI is especially sensitive to motion ^24^. A recent study showed that small head movements that do not cause visible artifact in structural brain images can also produce spurious reductions in estimated gray matter volume or cortical thickness ^25^.

Many studies provide minimal information about the specific methods used to control for movement in a patient population that by definition is going to exhibit more movement than the average subject. Therefore inadequate control of subject movement may have contributed to some of the inconsistent results in past neuroimaging studies.

### Phenomenology and natural history

***The urge made me do it.*** Premonitory urges have been considered to have an important role in tic generation, and CBIT includes using a competing response to prevent a tic from occurring until the urge decreases sufficiently so that the tic will not occur. A number of articles in 2014 addressed how premonitory sensations and urges relate to tics and tic suppression.

Capriotti *et al.* ^33^ examined the effects of negative reinforcement on premonitory urges in 13 children and adolescents with TS or chronic tic disorder (CTD). Subjects rated their urges to tic during three conditions: baseline during which they freely ticced, reinforced tic suppression and reinforced tic suppression with escape. During the escape condition, subjects could initiate a 10 second break during which they could freely tic. When the break was over, the reinforced tic suppression began again. Tic rates were significantly lower during reinforced suppression conditions compared to baseline free-to-tic conditions, although tic rates were significantly higher during the breaks in the escape condition compared to non-break periods. Urge ratings were significantly higher during the reinforced tic suppression conditions compared to the baseline periods and in the escape condition, urge intensity went down from break onset to the end of a break. These results support the hypothesis that premonitory urges are maintained through a process of negative reinforcement.

Many people with tics say that they perform tics to decrease the intensity of premonitory urges because the urges are so bothersome. The relationship between feelings of discomfort and habituation was studied in 90 healthy undergraduate humans with no tic diagnosis ^34^. A 2×2 experimental design was used with subjects either receiving an air puff to the eye or hearing a sound, and either receiving an instruction to blink or no instruction to blink. When subjects received the air puff and instructions to blink, the air puff was less annoying but the EMG response of the orbicularis oculi muscle continued and the length of the EMG response actually increased. When subjects received the air puff without any instructions about blinking, habituation to the air puff occurred. These results indicated that blinking was reinforced by the decrease in annoyance and yet this process also prevented habituation from occurring. A similar process may establish the association between premonitory urges and tic behaviors; if so, this study may provide an interesting “animal” model of tics for certain studies.

Treatment-naive children and teenagers with chronic tic disorders were compared while being allowed to tic freely and while receiving reinforcement for suppressing their tics ^35^. Attentional difficulties and age did not affect ability to suppress tics. Interestingly, subjects were able to suppress tics associated with more intense urges just as much as tics associated with less intense urges.

The Premonitory Urge for Tics Scale (PUTS) ^36^ has been the primary instrument for evaluating premonitory urges in children and adolescents. A 9 item version is frequently used because one item (“I am able to stop my tics, even if only for a short period of time”) did not correlate well with other test items. Interest in determining the reliability and validity in older adolescents and adults produced several studies that were published in 2014. The 10-item PUTS was completed by 102 adults at two specialist clinics. Again item 10 demonstrated relatively low item-total correlation, consistent with the idea that tic suppression and premonitory urges may reflect different mental processes ^37^. The PUTS total score correlated only slightly with scores on the Motor tic, Obsessions and Compulsions, Vocal tic Evaluation Survey (MOVES) (total 0.34, motor 0.28, vocal 0.27), supporting the view that tics and premonitory urges may involve different processes. In general, however, the PUTS was considered to have acceptable reliability and validity when used with adults. Another study examined PUTS scores in 122 older adolescents and adults with TS or CTD ^38^. A third study examined the use of the PUTS in 100 adults with TS ^39^. PUTS scores were related to obsessive-compulsive symptoms, anxiety, attentional problems and quality of life. Half of the total sample had “pure” TS while the other half had comorbid conditions (including 23 with OCD, 15 with ADHD, and 6 with anxiety). For patients with “pure” TS, premonitory urges were negatively related to quality of life scores while a weaker relationship was seen between these two variables for patients with comorbid conditions. When stepwise multiple linear regression analyses were performed, PUTS scores for the “pure” subgroup were only predicted by MOVES obsessive-compulsive subscale scores, while for the subgroup with comorbidities only anxiety scores were predictive of premonitory urges.

At this time the PUTS is the only empirically validated measure of premonitory urge severity. However, Capriotti *et al.* pointed out that the PUTS is relatively insensitive to change and is of limited validity in children under the age of 10 ^33^. They suggested the number of breaks taken during the tic suppression reinforcement + escape trials as an alternative way of measuring premonitory urge intensity. New approaches to quantifying urge intensity would be welcome.

***What generates and maintains tics?*** Two stress-induction tasks ( *i.e.*, public speech, discussion of family conflict) were used to study 8 TS children with comorbid anxiety ^40^. Tic frequency did not increase during periods of increased heart rate, and during the public speech task tic frequencies were actually lower during periods of increased heart rate. The authors point out the only psychophysiological measure of stress used in this study was heart rate and that future studies may benefit from simultaneously assessing a variety of measures of stress (e.g., respiratory rate, ECG, eye tracking) and examining effects on premonitory urge intensity in addition to tic occurrence.

***They went away.*** Shprecher *et al.* reported a retrospective follow-up study of tic remission ^41^. A brief survey was used to assess current symptoms of 53 TS patients who were 13–31 years old and had been seen previously in a TS clinic. At the time of the follow-up subjects were seen in person or contacted by telephone. The survey results were consistent with past research about TS and comorbid ADHD and OCD. Mean symptom onset was age 7.9 for both tics and ADHD and 9.2 for OCD. Peak symptom severity was reported to be around age 11–13 for all three conditions with a decline in symptom severity beginning around age 14–15. Symptom remission was reported in 32%, 23%, and 21% of subjects for tics, ADHD, and OCD respectively.

Limited longitudinal follow-up data are available for tic disorders other than TS. Bisker and colleagues reviewed 43 children with no prior diagnosis of Tourette syndrome who had been diagnosed with ocular tics by a pediatric neuro-ophthalmologist ^42^. An average of 6 years after their initial consultation, 32 of the children were located for follow-up. Of these, 44% had persistent ocular tics, 9% had developed nonocular motor tics, and 16% had developed both nonocular motor tics and vocal tics. In other words, the tic disorder remitted in less than a third of the patients available for follow-up.

### Treatment

***Medication.*** The effectiveness of dopamine D2-like receptor antagonists for treating tics is well-established ^46, 72^. Recently ecopipam, a selective D1 receptor antagonist, was used in an 8-week open-label study in 18 TS adults, 15 of whom completed the study ^73^. The mean YGTSS total tic score decreased from 30.6 at baseline to 25.3 at the end of the trial. There was no worsening of ADHD or OCD symptoms. Interestingly, there was no change in the intensity of premonitory urges as measured by the PUTS scale, suggesting that patients may have felt more able to resist the urges to perform tics. There was no weight gain associated with taking ecopipam, as is seen with most D2 antagonists. The results of this pilot study suggest the value of conducting a larger double-blind study. Given that this medication has a different mechanism of action than the medications currently available to treat tics, it may prove useful for the nontrivial number of patients who have responded inadequately to currently available medications.

Wijemanne *et al.* ^74^ provide a retrospective chart review of patients treated with fluphenazine at a movement disorders referral center over a 26-year period. Fluphenazine is a high-potency typical antipsychotic. Only patients who had at least one follow-up office visit or telephone interview were included in the study. A total of 268 patients were included in the study; they had taken a mean daily fluphenazine dose of 3.24 mg for an average of 2.6 years. Improvement was judged to be moderate to marked in four fifths of patients, with side effects in one fourth. Tardive dyskinesia was not observed.

***Deep brain stimulation.*** Deep brain stimulation (DBS) is a promising treatment option for highly selected TS patients refractory to more conservative treatments ^50, 75^. Several reports published in 2014 provide open-label follow-up on deep brain stimulation (DBS) in more than a few TS patients. Several groups had targeted the internal segment of the globus pallidus (GPi) ^76– 78^. At follow-up intervals of from 8 to 80 months, most patients tolerated the treatment well and mean symptomatic improvement compared to pre-surgery was 40–50%. Another group reported open-label 6- and 12-month follow-up of 8 patients after DBS to the ventral anterior and ventrolateral thalamus ^79^.

Angelov and colleagues ^80^ studied DBS in rats bred for high prepulse inhibition (PPI) of the startle reflex, an electrophysiological marker that is elevated in TS and some other illnesses. This allowed direct comparison of results (in terms of PPI reduction) among several different previously proposed target sites for DBS in TS. They found that thalamic stimulation (of the centromedian-parafascicular complex) best reduced PPI and nucleus accumbens less so. Importantly, implantation of leads in the entopeduncular nucleus (the rodent homolog of the primate GPi) reduced PPI, but DBS itself added no additional benefit; this result helps demonstrate why randomized, blinded studies are crucial in evaluating invasive treatments.

***Behavior therapy really works.*** Behavior therapy has been studied as a treatment for tics for many years ^26^. However, its adoption in clinical practice has lagged for a number of reasons ^27^. A meta-analysis that appeared in 2014 may help convince skeptics of its efficacy. The meta-analysis included 8 randomized control trials of TS behavior therapy with a total of 438 TS subjects ^28^. There was no evidence of publication bias. Treatment effects were in the medium to large range, with a number needed to treat (NNT) of only 3, comparable to the most effective class of medications (antipsychotics). Participants who were more likely to respond to behavior therapy were older, had more therapeutic contact and were less likely to have comorbid ADHD. At this point, the evidence base for behavior therapy’s efficacy in treating tics is stronger than for any other class of treatments except antipsychotics ^26^.

Although research continues to demonstrate the value of behavioral treatments such as CBIT, a limited number of therapists have been trained to administer CBIT. Given the distance that many patients live from potential therapists there is a need for alternative forms of treatment. Blount *et al.* reported on treatment administered using an intensive outpatient procedure (i.e., several hours daily over a four day period) to two pediatric outpatients. This treatment resulted in significant tic reductions that were maintained at follow-up 6 to 7 months later ^29^. This form of treatment may be more convenient for patients and their families who need to travel a significant distance for treatment, but a randomized controlled trial will be needed to replicate these promising results.

***Do exercise and biofeedback work?*** A small group of 18 participants, ranging in age from 10 to 20 years old, performed an Xbox ^®^ kickboxing exercise routine with a 5-minute easy exercise session followed by a 2-minute break and then a 5-minute exercise session that was more physically demanding ^30^. Tic counts based on video recordings were lower during both exercise sessions compared to during a baseline interview ( *i.e.*, completion of the Physical Activity Questionnaire for Adolescents, discussion about hobbies and other leisure activities). Interestingly, tic frequency was higher during the more demanding exercise session (which also occurred after the subjects had been exercising for a longer amount of time) than during the easier exercise session. Although tic frequency increased significantly during a second interview completed about 30 minutes after the end of the exercise sessions, the frequency was still below that seen during the pre-exercise baseline. Exercise also resulted in significantly reduced self-reported anxiety which was maintained during the post-exercise interview. It was suggested that exercise might have been effective in reducing tic frequency because it improved executive control functions, or because it served as a distraction task taking attention away from the tics, or because exercise functioned as a competing response which made it more difficult to perform the tics. Behavioral treatments, such as CBIT, tend to involve multiple components and, consequently, it is difficult to determine whether all components are necessary for all patients. Using a simple intervention such as that used by Nixon *et al.* may make it easier to identify the underlying mechanism that makes the intervention effective and may help identify whether certain patient subgroups are more likely to respond to a particular treatment component.

A preliminary randomized controlled trial examined electrodermal biofeedback during three 30-minutes sessions each week for 4 weeks in 21 adults with TS ^31^. Both sham and actual biofeedback produced similar decreases in tic frequency and similar improvements in well-being. The authors noted that tics occurring during the biofeedback sessions resulted in competing phasic electrodermal arousal responses making it difficult for patients to sustain a reduction in sympathetic tone, suggesting that modifications in the treatment protocol might increase effectiveness. The sham procedure, which involved providing feedback to subjects so that they thought that they were successfully altering their electrodermal activity, also resulted in a significant decrease in tics. This is surprising since placebo effects are minimal in pharmacological trials ^32^.

### Old and new

The most recent International Scientific Symposium on Tourette Syndrome (New York, 2009) led to a set of review articles on TS updated for publication in 2014 ^27, 43– 50^. Also in 2014 the Tourette Association of America announced that it had joined with two European TS groups to sponsor the “First World Congress on Tourette Syndrome and Tic Disorders”, held in London in June, 2015 ( http://touretteworldcongress.org). Finally, it is difficult to resist pointing out that 2014 also marked the introduction of a publication channel devoted entirely to tics, *F1000Research: Tics* ^51^. New submissions are warmly invited!

## Discussion

We have provided summaries of some of the articles published in 2014 that we think will contribute to further advances in the field. They cover a variety of topics: genetics, animal models, neuroimaging, and pharmacological and nonpharmacological treatment. The choice of articles was admittedly subjective and most likely incomplete; in fact, we have listed a few more papers in Box 1. However, one of the beauties of this publication venue is that readers who feel we have misjudged are welcome to add their own recommendations to the comments section of this article online.

We look forward to reprising this “highlights” page at the end of 2015, and would be grateful for article nominations or other suggestions from readers. Box 2 starts off this process by listing some meeting presentations and preprints that caught our interest but had not appeared in final form by the end of 2014. We hope that 2015 brings important breakthroughs in our understanding of the causes, mechanisms and treatment of tic disorders.

Box 1. **Additional 2014 publications of interest**

“Altered synaptic plasticity in Tourette’s Syndrome and its relationship to motor skill learning” ^52^

“Environmental circumstances influencing tic expression in children” ^53^

“The modulating role of stress in the onset and course of Tourette’s Syndrome: A review” ^54^

“Tic-related obsessive-compulsive disorder (OCD): Phenomenology and treatment outcome in the Pediatric OCD Treatment Study II” ^55^

“Set-shifting deficits: A possible neurocognitive endophenotype for Tourette Syndrome without ADHD” ^56^

“Variables associated with tic exacerbations in children with chronic tic disorders” ^57^

Prenatal and perinatal risk factors for TS ^58^

Prenatal risk factors for TS ^81^

“Tics are caused by alterations in prefrontal areas, thalamus and putamen, while changes in the cingulate gyrus reflect secondary compensatory mechanisms” ^59^

“Meta-cognitions in Tourette syndrome, tic disorders, and body-focused repetitive disorder” ^60^

“Dysregulated intracellular signaling in the striatum in a pathophysiologically grounded model of Tourette syndrome” ^61^

Box 2. **Work to look for in 2015**

“Don’t look”: seeing your own tics makes them more frequent ^62^

Astrocyte metabolism in TS ^70^

Functional connectivity and machine learning in TS ^63^

Reward enhances tic suppression very early in the course of tic disorders ^64^

Transcriptome analysis of the human striatum in Tourette syndrome ^65^

Influence of gender on Tourette syndrome beyond adolescence ^66^

Attention and tic suppression in TS ^67^

Mindfulness-based stress reduction ^68^

Ablation of striatal cholinergic interneurons ^10^

Deep brain stimulation for TS ^75^

Deep repetitive transcranial magnetic stimulation to the supplementary motor area ^82^

Neurofeedback and TS ^83^

Psychiatric comorbidity in TS ^84^

Current medication treatment of TS in Germany ^85^

Parenting stress in TS ^86^

The Tourette Association of America’s links to scientific articles of interest ^87^

## Data availability

The data referenced by this article are under copyright with the following copyright statement: Copyright: © 2015 Richards CA and Black KJ

Data associated with the article are available under the terms of the Creative Commons Zero "No rights reserved" data waiver (CC0 1.0 Public domain dedication). 

F1000Research: Dataset 1. Publications on Tourette syndrome: 1950–2014., 10.5256/f1000research.6209.d79935 ^71^

## Supplementary materials

**IPython notebook.** This is an IPython notebook file for anyone who wants to recreate or update Figure 1.

.

http://dx.doi.org/10.5256/f1000research.6209.s79933

**Same notebook in HTML.** The same file for viewing in a web browser, for readers who do not use IPython.
