# Supplementary material for: Tourette Syndrome research highlights 2014
Source: F1000Res. 2015 Jul 14;4:69. Originally published 2015 Mar 16. [Version 2] doi: 10.12688/f1000research.6209.2 (PMC4602330; doi:10.12688/f1000research.6209.2)
Supplement: Supplementary file 3 [file f1000research-4-7260-s0002.tgz › 688868f3-c332-4a6f-b16c-df036fc816b9.html]

Notebook


**Figure 1. Publications on Tourette syndrome.** The number of new publications on Tourette syndrome or other tic disorders each year was estimated from PubMed. The colored line is a LOWESS (locally weighted scatterplot smoothing) curve from the primary data.

PubMed was searched on 29 Jun 2015 using the search string “`("Tic Disorders"[MeSH] OR Tourette NOT Tourette[AU]) AND datestring`” for various values of `datestring`. For `datestring=1800[PDAT]:1950[PDAT]`, the search returned zero articles. Subsequent searches used `datestring=year[PDAT]:next[PDAT]` for each year from 1950 through 2014, where `next` means `year + 1`. (This strategy addresses PubMed’s listing both electronic and subsequent paper publication dates for about 250 TS publications since 2005.) The graph was generated by matplotlib in python (see supplementary material).

In [1]:

```
f1000research_color=tuple([value/255.0 for value in (203,97,45)])
f1000research_h1   =tuple([value/255.0 for value in (55,67,76)])
f1000research_h2   =tuple([value/255.0 for value in (33,97,128)])
```

In [2]:

```
import numpy as np
import csv
with open('TS_publications_20150629.csv', 'rb') as datafile:
    data2 = np.recfromcsv(datafile, names=True)
years = data2['year'][0:-1]
pubs  = data2['pubsfinal'][0:-1]
```

In [3]:

```
import statsmodels.api as sm
b = sm.nonparametric.lowess(pubs,years,frac=1./3)

import matplotlib.pyplot as plt
%matplotlib inline
plt.scatter(years[1:],pubs[1:],color='black')
plt.plot(b[1:,0],b[1:,1],color=f1000research_color,linewidth=3)
plt.axis((1950,2015,0,250))
plt.xlabel('Year',fontsize=16)
plt.ylabel('Publications (PubMed)',fontsize=16)
plt.legend(['smoothed','new publications'], loc='upper left')
plt.savefig('TS_publications_2014_v2.png', dpi=600, transparent=True)
plt.show()
```

In [4]:

```
print "year\tpubs" ; print "====\t===="
for index in range(0,years.size):
    print "{0:>}\t{1:>4}".format(years[index],pubs[index])
```

```
year	pubs
====	====
1950	   0
1951	   1
1952	   0
1953	   0
1954	   0
1955	   0
1956	   0
1957	   1
1958	   0
1959	   0
1960	   0
1961	   4
1962	   3
1963	   1
1964	   5
1965	  13
1966	  26
1967	  33
1968	  29
1969	  27
1970	  30
1971	  29
1972	  45
1973	  40
1974	  40
1975	  27
1976	  36
1977	  38
1978	  43
1979	  56
1980	  50
1981	  56
1982	 101
1983	  71
1984	  84
1985	  75
1986	  96
1987	  85
1988	 104
1989	  76
1990	  91
1991	  87
1992	 142
1993	 104
1994	 114
1995	 120
1996	 112
1997	 144
1998	 102
1999	 111
2000	 102
2001	 167
2002	 117
2003	 141
2004	 151
2005	 140
2006	 179
2007	 151
2008	 172
2009	 187
2010	 195
2011	 188
2012	 180
2013	 242
2014	 208
```

In [4]:

```

```
