# Supplementary material for: Tourette Syndrome research highlights 2014
Source: F1000Res. 2015 Jul 14;4:69. Originally published 2015 Mar 16. [Version 2] doi: 10.12688/f1000research.6209.2 (PMC4602330; doi:10.12688/f1000research.6209.2)
Supplement: Peer review history

# Peer review: Referee response for version 2

**DOI:** 10.5256/f1000research.7260.r10564

**Published:** 2015-09-28

**Authors:** Davide Martino

**Affiliations:** 1 Department of Neurology, King's College London, Hospital NHS Foundation Trust, London, UK

I have reviewed the revised version and the Authors' reply to my comments. I am pleased to say that the manuscript has now my full approval.

I have read this submission. I believe that I have an appropriate level of expertise to confirm that it is of an acceptable scientific standard.

# Peer review: Referee response for version 2

**DOI:** 10.5256/f1000research.7260.r9783

**Published:** 2015-08-04

**Authors:** Valerie C. Brandt

**Affiliations:** 1 Department for Pediatric and Adult Movement Disorders and Neuropsychiatry, Institute of Neurogenetics, University of Lübeck, Lübeck, Germany

The authors summarize papers, which they felt were notable or might inspire further research.

Strengths of the manuscript: the paper provides a well-written, informative and succinct summary of progress in a wide range of research areas concerning TS.

Weaknesses: the paper reports on research highlights 2014 and it should therefore be clearly stated in every section why this particular work is important for the field, this is not always the case (see comments below). I feel that not all sections are entirely clear without having read the original article; I therefore have a few minor suggestions (see below).

*General comment:* some papers that were published prior to 2014 are cited (e.g. the histidine findings), others are not (e.g. enhanced cognitive control).

*Specific comments:*

**A genetic clue:**

It would be interesting to include a) how precise the prediction of the diagnosis was, b) which SNP survived correction for multiple tests and c) possibly the authors main thoughts on the role of the nearby genes in TS.

**Pathophysiology:**

How large is the family (N)? Were dopamine receptor binding and prepulse inhibition altered in the same direction in humans and mice? What are the implications of the results for TS in general or possibly for a sub-group, i.e. why are they so important for the field?

**Other animal models:**

What are the implications of the McCairn paper, especially regarding vocal tics?

**Neuroimaging studies**:

“Cortical structure BOLD signal clearly preceded signal in subcortical structures“ - It may be interesting to point out that the temporal pattern of activation differs from animal models ( Bronfeld *et al*., 2013).

The paragraph about interhemispheric balance and role of SMA is very interesting and it would be nice to give a few more details regarding the model. The reference regarding induced echophenomena is missing.

**GABA in SMA study**: the findings are very complex and not easy to follow. It is unclear what sort of task the fMRI BOLD response refers to. It may also be useful to point out that fractional anisotropy in the corpus callosum was measured in fibers connected to the SMA and to point out why altered FA in CC might be related to increased motor tic severity and at the same time increased GABA in the SMA.

References for enhanced volitional control are missing.

**CBIT study**

Please include the behavioral findings.

**The urge made me do it**

The section has a very informative introductory sentence. However, the negative reinforcement model is explained in the third paragraph, after research on the model has already been presented. It might be useful to explain it in the introductory sentence.

**PUTS**

The presented results do not explain how the paper arrived at the conclusion that the PUTS has acceptable reliability and validity.

Results concerning MOVES associations with PUTS in the third study seem to contradict results found in the first study. Is there an explanation as to why that might be the case?  Are there noteworthy results found in the second study or do the results in the last paragraph refer to the second AND third study?

I have two more suggestions for Box 1:

Muller-Vahl, K. R., Riemann, L., & Bokemeyer, S. (2014). Tourette patients' misbelief of a tic rebound is due to overall difficulties in reliable tic rating. J Psychosom Res, 76, 472-476.

Gharatya, A., Stern, J., Man, C., Williams, D., Simmons, H., & Robertson, M. (2014). Suicidality in patients with tourette's syndrome. J Neurol Neurosurg Psychiatry, 85, e3.

I have read this submission. I believe that I have an appropriate level of expertise to confirm that it is of an acceptable scientific standard, however I have significant reservations, as outlined above.

# Response

**Published:** 2015-08-06

**Authors:** Kevin J Black

**Affiliations:** Department of Psychiatry, Washington University in St. Louis, USA

We thank Dr. Brandt for her thoughtful comments.

# Peer review: Referee response for version 1

**DOI:** 10.5256/f1000research.6659.r7989

**Published:** 2015-04-29

**Authors:** Barbara Coffey

**Affiliations:** 1 Department of Psychiatry, Icahn School of Medicine at Mount Sinai, New York, NY, USA

The authors report on highlights of the growing medical and scientific literature on Tourette Syndrome in 2014. The aim was not to conduct a systematic review, but instead to discuss noteworthy articles of interest. Topics ranged from animal models and neuroimaging to behavioral treatment.

Strengths of this manuscript were 1) cogent and thoughtful summary/discussion of the articles, 2) an informal and readable style, and 3) interesting content. There were several weaknesses, which if addressed, would strengthen the manuscript.

Methods: it would be helpful to know the approximate denominator of articles reviewed, and what proportion were selected.

Results: it would be helpful to know how/why topic areas were chosen. Weight seemed to lean strongly toward neuroimaging, which may reflect the authors’ primary interests and expertise. Organization of topic areas could be improved; as it stands, the reader is moved from neuroscience (animal models, imaging) to behavior therapy, premonitory urges, and longitudinal course, and then back to neuroscience (genetics). It might read more easily to start with basics (neuroscience, genetics, neuroimaging) through phenomenology (course, urges, role of stress) to treatment.

The manuscript could be improved with the inclusion of pharmacotherapy updates; although the Discussion summarized that the “broad spectrum of articles” covered “animal models, neuroimaging, and pharmacological and non-pharmacological treatment,” the only treatments discussed were behavior therapy, exercise and biofeedback.

Suggested pharmacotherapy additions include:

Gilbert, D. *et al.: *“ A D1 receptor antagonist, ecopipam, for treatment of tics in Tourette Syndrome” Clin Neuropharmacol 2014; 37 (1) 26-30

Malaty, I.A. and Akbar U: Updates in Medical and Surgical Therapies for Tourette Syndrome; 2014; Curr Neurol Neurosci Rep 14: 458; this is a review with an annotated bibliography.

Work to look for in 2015: Bachmann, CJ *et al.:* Trends in Psychopharmacological Treatment of Tic Disorders in Children and Adolescents in Germany; Eur Child Adolesc Psychiatry 2015; 24 (2); 199-207

Lastly, there appeared to be a relative lack of articles on aspects of psychiatric comorbidity. Of particular interest is the recently published study from the Tourette Syndrome Association International Consortium for Genetics:

Hirschtritt, M. *et al*.: Lifetime Prevalence, Age of Risk, and Genetic Relationships of Comorbid Psychiatric Disorders in Tourette Syndrome; *JAMA Psychiatry *doi:10.1001/jamapsychiatry.2014.2650

I have read this submission. I believe that I have an appropriate level of expertise to confirm that it is of an acceptable scientific standard, however I have significant reservations, as outlined above.

# Response

**Published:** 2015-06-30

**Authors:** Kevin J Black

**Affiliations:** Department of Psychiatry, Washington University in St. Louis, USA

We thank Dr. Coffey for her very thoughtful comments, and have revised the manuscript accordingly. Replies to specific comments follow.

- **Requests the “approximate denominator of articles reviewed.”**
  We read at least the abstract from the 200 papers identified in the PubMed searches. We have added this number to the introduction. We cite well over 50 of them. Originally we intended to discuss only about 5 or 10 papers, but it was harder than expected to omit anything.
- **“It would be helpful to know how/why topic areas were chosen … may reflect the authors’ primary interests”**
  Doubtless the selection of articles reflects some influence of our own interests. As you noted, we at least warned the reader that our selection was arbitrary. We attempted to respond to the theme of what reports were most likely to be of greatest interest in the future, and we now explain this choice in the first sentence of Results.
- “ **Organization of topic areas could be improved”**
  Thank you for the useful suggestion. We have rearranged the topics in a more structured fashion: Etiology, pathophysiology, phenomenology and natural history, and treatment.
- **We left out pharmacology**
  We agree. Please see our comments in the response to Dr. Martino's review about Gilbert *et al. *We have added the Malaty and Akbar citation to the treatment section, and the Bachmann *et al. *review to Box 2.
- **“Relative lack of articles on … psychiatric comorbidity.” Recommends Hirschtritt *et al. *2015.**
  We are also very interested in psychiatric comorbidity, and we agree that the Hirschtritt et al article is very important. As it was published in April, 2015, we added it to Box 2.

# Peer review: Referee response for version 1

**DOI:** 10.5256/f1000research.6659.r8212

**Published:** 2015-04-21

**Authors:** Davide Martino

**Affiliations:** 1 Department of Neurology, King's College London, Hospital NHS Foundation Trust, London, UK

I enjoyed reading this useful and well written contribution.

I have a few comments:

1. In the subsection of Results entitled “Other animal models”, the reader might benefit from a brief explanation of the model presented by McCairn et al: did this use a GABA antagonist like bicuculline?
2. In the subsection entitled “A look inside: neuroimaging studies”, I personally found interesting a paper which is not cited and evaluates the potential of deep repetitive transcranial magnetic stimulation (deep rTMS) to the supplementary motor area ( Bloch *et al*.,2014)
3. In respect to neurobiofeedback, as commented on in page 4, another useful paper on ADHD and tic disorders is Gevensleben *et al. *(2009). Moreover, a 2015 citation focusing on neurofeedback is this useful review: Farkas *et al*. (2015).
4. My main criticism is that there is no citation at all about deep brain stimulation, which is an area currently avidly investigated in Tourette syndrome. It is possible that the authors felt that none of the articles published in 2014 on DBS in Tourette syndrome is sufficiently interesting, but I would at least discuss this in the article and add the following citation in Box 2: Schrock *et al*., (2015).
5. Additional 2014 publications of interest:
Chao TK, Hu J and Pringsheim T: Prenatal risk factors for Tourette syndrome: a systematic review. *BMC Pregnancy and Childbirth. *2014; **14**(53).
Gilbert DL, Budman CL, Singer HS, *et al*:, A D1 receptor antagonist, ecopipam, for treatment in Tourette syndrome. *Clin Neuropharmacol. *2014; **37**(1): 26-30.
Wijemanne S, Wu LJ and Jankovic J: Long-term efficacy and safety of fluphenazine in patients with Tourette syndrome. *Mov Disord. *2014; **29**(1):126-130.

I have read this submission. I believe that I have an appropriate level of expertise to confirm that it is of an acceptable scientific standard, however I have significant reservations, as outlined above.

# Response

**Published:** 2015-06-30

**Authors:** Kevin J Black

**Affiliations:** Department of Psychiatry, Washington University in St. Louis, USA

We appreciate Dr. Martino's thoughtful comments and have used them to improve the manuscript for version 2.

1. **Brief explanation of the model presented by McCairn *et al***.
  Yes, this used bicuculline injected into the putamen or nucleus accumbens; we have added this to the manuscript.
2. **Recommends Bloch *et al***.
  This is a very interesting article, but the final version was not yet published as of May, 2015. We have added it to Box 2.
3. **"In respect to neurobiofeedback, as commented on in page 4, another useful paper on ADHD and tic disorders is Gevensleben *et al.* (2009). Moreover, a 2015 citation focusing on neurofeedback is this useful review: Farkas *et al*. (2015)."**
  Good articles. Readers can find the citation to Gevensleben *et al. *2009 in your comments. We have added the Farkas *et al. *(2015) reference to Box 2.
4. **No citation about DBS.**
  We agree this is an important area of work in TS, but relatively few DBS papers on TS were published in 2014. We have added a paragraph on DBS, and have added the important 2015 paper you suggested to Box 2.
5. **Recommends Chao *et al.*, prenatal risk factors for Tourette syndrome**
  We agree and have added this to Box 1.
**Recommends Gilbert *et al.*, ecopipam study**
  This paper was overlooked by accident. We agree this was an important pilot study on a potentially new avenue of treatment for TS and have added a short paragraph.
**Recommends Wijemanne *et al.*, fluphenazine report.**
  Thank you. We have added this to the section on medication treatment.
